# Light-based therapy of infected wounds: a review of dose considerations for photodynamic microbial inactivation and photobiomodulation

**DOI:** 10.1117/1.JBO.30.3.030901

**Published:** 2025-02-07

**Authors:** Nidhi Singh, Lothar Lilge

**Affiliations:** a University of Toronto, Department of Medical Biophysics, Toronto, Ontario, Canada; b Sunnybrook Research Institute, Toronto, Ontario, Canada; c University Health Network, Princess Margret Cancer Centre, Toronto, Ontario, Canada

**Keywords:** antimicrobial photodynamic therapy, photobiomodulation, wound healing, planktonic, *in vitro*, *in vivo*

## Abstract

**Significance:**

Chronic or surgical wound infections in healthcare remain a worldwide problem without satisfying options. Systemic or topical antibiotic use is an inadequate solution, given the increase in antimicrobial-resistant microbes. Hence, antibiotic-free alternatives are needed. Antimicrobial photodynamic inactivation (aPDI) has been shown to be effective in wound disinfection. Among the impediments to the wide utility of aPDI for wounds is the high variability in reported photosensitizer and light dose to be effective and unintentional detrimental impact on the wound closure rates. Additionally, the time required by the healthcare professional to deliver this therapy is excessive in the present form of delivery.

**Aim:**

We reviewed the dose ranges for various photosensitizers required to achieve wound disinfection or sterilization while not unintentionally inhibiting wound closure through concomitant photobiomodulation (PBM) processes.

**Approach:**

To allow comparison of aPDI or PBM administered doses, we employ a unified dose concept based on the number of absorbed photons per unit volume by the photosensitizer or cytochrome C oxidase for aPDI and PBM, respectively.

**Results:**

One notes that for current aPDI protocols, the absorbed photons per unit volume for wound disinfection or sterilization can lead to inhibiting normal wound closure through PBM processes.

**Conclusion:**

Options to reduce the dose discrepancy between effective aPDI and PBM are discussed.

## 1 Introduction

Wounds caused by chronic health conditions, accidental or intentional by surgical interventions, present a tremendous direct and indirect cost burden to the healthcare systems and the patients, respectively. In 2021, the global wound care market was valued at 20.59 billion USD, and it is projected to expand at a compounded annual growth rate of over 4% until 2030. The rise in surgical procedures and chronic disease prevalence drives wound care products’ growth. The North American fraction of the direct healthcare costs exceeded 9 billion in 2021.^1^ The impact of direct and indirect costs for wound management also differs between high versus middle- and low-income environments. Healthcare costs and projections in some societies are increasing at much faster rates, for example, in the case of Singapore.^2^ Historically, critical colonization implied a microbial burden of $10^{5}\;\mathrm{CFU}\;g^{-1}$ of wound tissue being associated with delayed healing,^3^ but the synergy of wound microorganisms, their virulence, and quantity play significant factors in delaying wound healing.^4^ This leads to critical colonization being associated with biofilm infection. Hence, targeting both the microorganisms and the biofilm is required.

Surgical site infections (SSIs) are the most expensive hospital-acquired infections (HAIs), accounting for 20% of all HAIs. It is estimated that SSIs result in $3.5 to 10 billion healthcare costs per year and extend the hospital stay by 7 to 11 days and increase the risk of mortality by 2 to 11 times in post-operative patients compared with patients without SSI.^5^ SSIs remain common for a range of cancer-related surgeries, ranging from a low single percentage in dermatologic surgeries in Germany^6^ to as high as 45% in head and neck cancer surgeries in India.^7^ A multi-center audit in the UK reported ∼16% of women with clinically diagnosed SSI after surgery for gynecological cancer. Of these women with SSI, 33% had prolonged hospital stays, and 29% who needed adjuvant chemo-therapy or radiotherapy had their therapy delayed.^8^ Olsen et al.^9^ reported an SSI incidence rate of 1.1% to 12.4 % in women undergoing breast surgery, depending on the surgery, with the highest incidence rate in mastectomy with immediate implant reconstruction. The attributed cost of SSI after breast surgery was reported at 4091 USD per patient. Sugamata et al.^10^ reported a 12.6% SSI incidence rate after laparoscopic resection of colorectal cancer. They reported a significantly lower post-operative relapse-free survival in patients with SSI (49.2%) compared with patients without SSI (87.2%), showing that SSI affects post-operative oncological outcomes in these patients.

To prevent local infections delaying wound closure, presurgical^11^ and perioperative antibiotic prophylaxis, particularly for SSI in organ transplant,^12^ have shown some cost benefit to the healthcare provider. Post-operative strategies for infection prevention^13^ include administration of silver-impregnated vacuum dressings, extended intravenous antibiotics, supplemental oxygen, ozone therapy,^14^ and nano-particle-based therapies.^15^^–^^17^ Microbes such as bacteria, fungi, and viruses can change or evolve upon exposure to antimicrobial therapy to evade the antimicrobial effect, resulting in resistance to these drugs. Antimicrobial resistance (AMR) is a serious global health issue that is placed in the top 10 public health threats by the World Health Organization. It is estimated that AMR can cost the global economy 7% of its gross domestic product (GDP) or 210 trillion USD by 2050.^18^ Of an estimated 5 million deaths associated with AMR infections worldwide, about 1.3 million deaths were directly attributed to AMR. About half of the 14,000 AMR-related deaths in Canada were caused directly by AMR strains. AMR-resistant strains are commonly encountered in patients with chronic wounds when topical or systemic antibiotics are administered regularly over prolonged periods, allowing new AMR strains to rise. This is of concern given that the prevalence of chronic wounds is increasing given that age and diabetes are two major risk factors and both are increasing in populations worldwide. The global tuberculosis (TB) report 2014^19^ reported 480,000 new cases of multi-resistant TB globally while the development of resistance to anti-malarial drugs and antiretroviral therapy is being monitored. Although TB and malaria are not factors in infected wounds, their incidence reduction is one of United Nations Educational, Scientific and Cultural Organization’s (UNESCO’s) sustainable development goals.^20^ A study of 217 infected wounds showed 28 species repeatedly, led by *Staphylococcus aureus* (37%), *Pseudomonas aeruginosa* (17 %), *Proteus mirabilis* (10%), *Escherichia coli* (6%), and *Corynebacterium* spp. (5%). The study also noted polymicrobial infection in 27% of the samples, with the most common combination comprising *S. aureus* and *P. aeruginosa*.^21^ Testing the patient-derived cultures against 17 antibiotics revealed that only Linezolid and Vancomycin were effective for all *S. aureus*, *Corynebacterium*, and coagulase-negative staphylococci. Other antibiotics do not affect 10% to 100% of the gram-positive bacteria. Reduced efficacy of 13 antibiotics against gram-negative *P. aeruginosa*, *P. mirabilis*, and *E. coli* was noted. None of the tested antibiotics showed adequate efficacy against all tested samples.^21^ Combating the increasing number of AMR bacterial strains requires reducing antibiotic prescriptions and developing other antimicrobial approaches, including peptides, cellulose, chitosan, and antimicrobial photodynamic inactivation (aPDI).

### 1.1 Antimicrobial Photodynamic Inactivation

aPDI, also called antimicrobial photodynamic therapy (aPDT) or photo antimicrobial chemotherapy (PACT), is based on the administration of a photosensitizer (PS) followed by exposure to light with a wavelength matching electronic energy transitions of the PS, resulting in it entering an excited triplet state, leading to the production of cytotoxic oxygen radicals, via either type I, hydroxy radicals, superoxide dismutase, and superoxide anion, following a charge transfer to water or type II initiated by energy transfer to molecular oxygen resulting in singlet oxygen or peroxide ions. The administration of exogenous PS for aPDI requires higher or faster accumulation in microbes versus mammalian host cells, which is attainable for short PS administration to light exposure time intervals in the 0- to 10-min range. Topical or local administration of PSs leads to their fast association with gram-positive and negative microbes and has shown high efficacy in controlling the infection and enabling accelerating wound healing.^22^ Conversely, allowing for prolonged PS administration to light exposure intervals was shown to be detrimental to wound healing. Tanaka et al.^23^ showed that delaying photoirradiation 24 h post Photofrin administration resulted in an Methicillin-Resistant Staphylococcus Aureus (MRSA) concentration increase in the knee joint attributed to excessive PDT-mediated neutrophil killing. aPDI has been applied to infected burns, incisions, or abrasion wounds in various pre-clinical and clinical situations, targeting the microbes directly or aiming to disrupt the biofilm-supporting microbes if present. The classes of PSs employed in antimicrobial and anti-biofilm PDT include porphyrins and porphyrin precursors, chlorins, other tetrapyrrols, and non-tetrapyrrols, as recently reviewed by Hu et al.^24^ The majority of the employed PSs have been approved for other medical indications, such as in oncology or image-guided surgery. They include methylene blue (MB), new methylene blue (NMB), rose bengal (RB), curcumin, toluidine blue O, Methyl-Aminolevulinic acid (Me-ALA) or ALA-induced Protoporphyrin IX (PpIX), and indocyanine green (ICG). These PSs are off-patent protection and commercially available, making them suitable for these predominately investigator-initiated research studies. One significant advantage of aPDI is its targeting not only the microbes but also the underlying biofilm to disrupt the microenvironment, protecting microbes and delaying recolonization in the case of chronically infected wounds.^25^ The clinical use of aPDI is advanced for periodontitis,^26^^–^^28^ the oral and nasal cavities for pre-emptive treatment, commonly relying on MB or ICG with their various derivatives as PS.^29^^–^^32^ Current attention is also paid to using aPDI as an antiviral therapy to reduce viral burden in the nasal cavity,^33^ currently applied in most hospitals throughout British Columbia, Canada, to patients prior to surgery. The efficacy of aPDI depends on the generation and maintenance of sufficiently high reactive oxygen species (ROS) concentrations to overcome the microbes’ natural protection against them. Microbial systems are well protected against $O_{2}^{•-}$ and $H_{2}O_{2}$ via superoxide dismutase and detoxification achieved by catalases and peroxidases. Still, they lack enzymatic protection against singlet oxygen, O21,^34^ and high concentrations of other ROS, which act by indiscriminately attacking lipids and proteins. Upon the absorption of a photon, the PS undergoes an “intersystem crossing” from the short-lived singlet excited state into the triplet excited state, allowing energy and/or spin exchange with ground state molecular oxygen, or water to generate singlet oxygen, O21 or it can gain electrons from nearby molecules to donate it either to oxygen, generating the ROSs mentioned above and hydroxyl radical ${\mathrm{HO}}^{•}$, or interacting with biomolecules as radical itself. Superoxide radical $O_{2}^{•-}$ can oxidize iron-sulfur clusters (${\mathrm{Fe}}_{4}S_{4}$) to dehydrate the mitochondria and cytosol, inactivating enzymes critical for aerobic metabolism in cells and releasing iron, which can further generate hydroxyl radical ${\mathrm{HO}}^{•}$ capable of oxidizing biomolecules.^34^ The generation of these short-lived radicals must proceed at high rates to overcome the microbe’s scavenging potential.

The cytotoxic dose rate, governed by the rate of ROS generation [Δg1](t), is given by the number of photons absorbed by the PS, determined by its concentration [C] and molar absorption coefficient, ε [$\mu M^{-1}\;{\mathrm{cm}}^{-1}$] at the treatment wavelength (λ); it ROS quantum yield, $\phi_{\Delta }$ here represented for singlet oxygen, O12 and the light energy density.

For surface applications such as debrided wounds, the photon density is determined by the light irradiance Φ [$\mathrm{mW}\;{\mathrm{cm}}^{-2}$]

[Δ1g](t)=ε(λ)[C]ΦϕΔ, (1)

and the number of absorbed photons is given by

[Δ1g](t)=ε(λ)[C]ΦhcλϕΔ, (2)

where h is Planck’s constant, and c is the speed of light in a vacuum. The total cytotoxic dose is given by the integral over the exposure time, here considering possible time-dependent changes of the PS concentration [C](t) due to photobleaching and other effects and temporal variations in the irradiance Φ(t) given by

∫[Δ1g](t)dt=∫ε(λ)[C](t)Φ(t)hcλΦΔdt. (3)

The integral represents the total O12 quantity, or cytotoxic moieties, generated due to the delivered radiant exposure, H, [$J\;{\mathrm{cm}}^{-2}$]. In deeply infected wounds or thick infected necrotic tissue, the attenuation of the photon density in depth must be considered. For large-area illuminations, the photon density as a function of depth, z, into the tissue decays exponentially, governed by the effective attenuation coefficient, $\mu_{\mathrm{eff}}$ [${\mathrm{cm}}^{-1}$]

$$\Phi (z,t)=\Phi (0,t)e^{-(\mu_{\mathrm{eff}}(\lambda )z)}.$$ (4)

The attainable concentrations of O21, [Δ1g], for aPDI in planktonic solution, *ex vivo* and animal studies are in the range of 100  μM to low mM and maintained over 100 s, overwhelming the defenses of the microbes, leading to their inactivation.

The efficacy of aPDI has been reviewed for various indications, including dentistry,^35^^,^^36^ water treatment,^37^ aquaculture,^38^ food supply,^39^^,^^40^ agriculture,^41^ implants,^42^^,^^43^ veterinarian medicine,^44^ burns,^45^ and chronic wounds.^46^^–^^48^

### 1.2 Photobiomodulation

Although the cytotoxic effect of aPDI is predominantly limited to the microbes, the aPDI excitation photons are also absorbed by the not-photosensitized mammalian host tissues, putative lead by cytochrome C oxidase (CCO)^49^ as primary chromophore, leading to a range of changes in the host’s signaling pathways affecting the cells metabolism and gene expression. These effects are commonly researched under the topic of photobiomodulation (PBM).

PBM, previously also known as low-level laser therapy or low-intensity laser therapy, uses only light to modulate biological processes in tissues. PBM is effective for various clinical conditions, including wounds, chronic pain, and reduction of lung and joint inflammation.^50^^–^^52^

CCO transfers an electron from cytochrome c to oxygen as part of the respiratory redox cycle. Studies showed that the wavelength-dependent PBM effects follow the absorption spectra of CCO with absorption peaks in the red and near-infrared (NIR) regions due to the presence of the heme group (heme a and heme b) and copper centers (CuA and CuB) in the enzyme.^53^^–^^55^ Heme has an extremely short and excited state lifetime and is photochemically inert. The two copper metal centers absorb both in their reduced and oxidized form, for CuA at 620 and 820 nm and for CuB at 760 and 680 nm, respectively.^56^^,^^57^ CCO is a dominant chromophore in aPDI beside hemoglobin and the PS, given its high molar extinction coefficient, ε, and high *in vivo* concentrations ranging from 6.5 mM (rat brains) to $70\;\mathrm{mg}\;g^{-1}$ dry weight in human skeletal muscle.^58^ Activation of the respiratory redox change by the added photon quantum energy results in the generation of Adenosine Triphosphate (ATP), ROS, and Nitric Oxi, which in turn results in the modification of gene expressions via the 5' Adenosine monophosphate-activated protein kinase (AMPK) and Protein kinase B (AKT) pathways.^57^

The PBM effect can be achieved at low irradiance with red or NIR wavelengths. Several factors affect the efficacy of treatment: irradiance, ϕ [$\mathrm{mW}\;{\mathrm{cm}}^{-2}$] radiant exposure, *H* [$J\;{\mathrm{cm}}^{-2}$], and illumination intensity modulation frequency, as well as repeatability. The World Association of Laser Therapy recommends limiting the delivered power density or irradiance to less than $100\;\mathrm{mW}\;{\mathrm{cm}}^{-2}$ and total energy density or *H* below $10\;J\;{\mathrm{cm}}^{-2}$. The majority of present studies evaluate radiant exposure as the driving PBM efficacy parameter. Higher ϕ and *H*, as employed for aPDI, have shown delays in uninfected wound closure speed in non-sensitized tissues^59^ as present on the basis of the wound at short times post-PS administration. *In vitro* studies demonstrated that irradiated fibroblasts from diabetic wounds survived 48 h better than unirradiated cultures following a single exposure of $5\;J\;{\mathrm{cm}}^{-2}$ of 660-nm irradiation.^60^ *In vitro* irradiating human vascular endothelial cells illuminated once with 808 nm resulted in a proliferation gain for up to 24 h, measured by scratch assay.^61^

Although most studies evaluated a positive effect of PBM on wound healing, they typically reported only on single radiant exposures Shoorche et al.^62^ reported the inhibition of Osteosarcoma’s migration capability and increasing cytoskeletal Young’s modulus for high irradiances or radiant exposures. Similarly, Rossi et al.^63^ reported decreasing metabolism and proliferation of fibroblasts for increasing *H*. Observing a beneficial biological response at low ϕ and *H* and an inhibitory response at high ϕ and *H* is commonly referred to as the biphasic effect in PBM, as coined by the group of Hamblin and others.^64^^–^^66^ PBM effects have been investigated at the molecular, histological, and functional levels for non-infected and infected wounds and need to be considered when evaluating aPDI efficacy. Pre-clinical studies have identified the wavelength ranges, λ, and optical parameters, including irradiance ϕ, radiant exposure *H*, and frequency of light irradiation, to attain high wound closure rates.

aPDI and PBM have both shown efficacy in the treatment of superficial infection and in aiding wound healing, respectively. As both are photonics-based techniques, there is a potential for significant interaction between the two relevant mechanisms and the development of a combinational therapy that uses aPDI for infection control and PBM for accelerated wound healing when managing ϕ and *H*, their respective wavelength or delivery sequence or frequency. Comparing and maximizing the efficacies of these two approaches poses challenges due to the insufficient reporting of experimental parameters in the existing literature.

Dick et al.^67^ proposed a consistent metric: the number of photons absorbed per unit volume to compare the results of *in vitro* photodynamic therapy studies and assess the reproducibility of the therapy. In this comprehensive review, we utilize this metric to establish a range of values within which aPDT can significantly inactivate bacteria in wounds, whereas PBM can effectively accelerate or not inhibit the wound-healing process. Wavelength and temporal separation of aPDT and PBM will be discussed to improve wound disinfection/sterilization with improved wound closure rates.

## 2 Methods

This review aims to identify trends in aPDI efficacy versus gram-positive and gram-negative bacteria as a function of treatment conditions under planktonic, preclinical, and clinical conditions while considering the applied ϕ and *H* with respect to the known biphasic tissue responses within the context of PBM. To render the efficacy of studies comparable even for disparate physical treatment conditions, concentration, and molar extinction coefficient of the PS and aPDI wavelength, λ, we calculated the number of photons absorbed by the PS per unit volume. We estimated the resulting ROS concentration or dose ([ROS]) for studies identified in the literature.

**Fig. 1:**
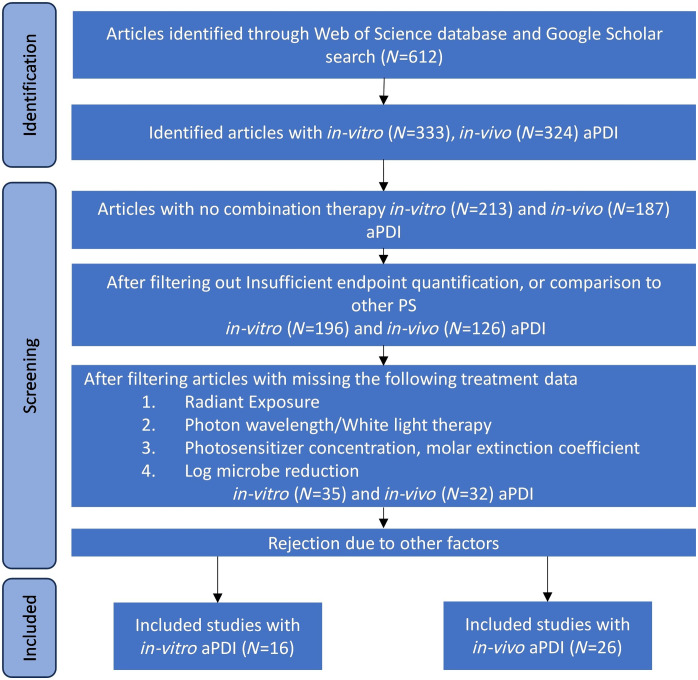
Literature selection process for aPDI in *in vitro* and *in vivo* studies.

Web of Science and Google Scholar databases were searched for the literature from 1997 to November 2024, inclusive. The literature included planktonic, preclinical, and clinical studies to evaluate aPDI efficacy and preclinical and clinical studies of PBM for wound healing. Search keywords included antimicrobial, photodynamic therapy, gram-positive, gram-negative bacteria, PBM, infected and non-infected wounds, *in vitro*, *in vivo*, and clinical studies. A total of 612 publications were initially scanned. Figure 1 shows the exclusion steps for the planktonic and *in vivo* preclinical and clinical studies. Only manuscripts providing either reduction in colony forming units (CFU) *in vitro* or *in vivo* or a rate of wound closure compared with controls were reviewed in more detail if they provided the information needed to calculate the absorbed photons to achieve the particular endpoints. See Fig. 1 for the selection process leading to the extracted studies.

*In vitro*, aPDI studies were restricted to those reporting the bacterial species, PS dose, λ, ϕ, exposure duration or *H*, and log microbe reduction. Data and sources for the PS’s molar extinction coefficients (ϵ(λ)) and ROS quantum yield ($\phi_{\Delta }$) are listed in Supplementary materials. Studies lacking any of the above parameters were excluded from the analysis as calculating the photons absorbed was impossible. A total of 41 datasets were extracted from 14 planktonic studies involving gram-positive bacterial species, and 53 were taken from 12 studies with gram-negative bacterial species for *in vitro* aPDI evaluation. Tables 1 and 2 show the studies that evaluated aPDI for inactivation of bacteria in planktonic solutions for gram-positive and gram-negative pathogens, respectively. From the data, we evaluated log reduction as a function of dose for all the datasets and gram-positive and gram-negative species separately. We evaluated the most frequently used dose range and for at least 3 log reduction in the pathogen population. We also looked at the log reduction as a function of wavelength and radiant exposure.

**Table 1:** Summary of aPDI data of gram-positive pathogens for studies in planktonic suspension and on biofilm.

S.No.	Ref	Pathogen	Photosensitizer	λ (nm)	Ext. coeff. (${\mathrm{cm}}^{-1}\;{\mathrm{mM}}^{-1}$)	Rad. exp. ($J\;{\mathrm{cm}}^{-2}$)	Photon abs. ($\mathrm{hv}\;{\mathrm{cm}}^{-3}$)	Log (photons abs. × QY)	Log red.
Gram-positive pathogens									
1	68	*Candida albicans*	Ce6	662	60.39	50.0	$4.02\cdot 10^{20}$	20.41	7.0
2	69	*C. albicans*	MB	660	71.55	8.0	$1.90\cdot 10^{20}$	19.97	1.0
3	69	*C. albicans*	MB	660	71.55	25.0	$5.94\cdot 10^{20}$	20.46	3.0
4	69	*C. albicans*	MB	660	71.55	40.0	$9.50\cdot 10^{20}$	20.67	5.0
5	69	*Enterococcus faecalis*	MB	660	71.55	0.7	$1.66\cdot 10^{19}$	18.91	1.0
6	69	*E. faecalis*	MB	660	71.55	2.0	$4.75\cdot 10^{19}$	19.37	3.0
7	69	*E. faecalis*	MB	660	71.55	4.0	$9.50\cdot 10^{19}$	19.67	5.0
8	69	*Enterococcus faecium*	MB	660	71.55	1.8	$4.28\cdot 10^{19}$	19.32	1.0
9	69	*E. faecium*	MB	660	71.55	5.0	$1.19\cdot 10^{20}$	19.76	3.0
10	69	*E. faecium*	MB	660	71.55	7.0	$1.66\cdot 10^{20}$	19.91	5.0
11	70	MRSA	ALA	650	5.05	384.0	$3.96\cdot 10^{20}$	20.44	7.0
12	71	MRSA	NB2	638	16.10	480.0	$2.48\cdot 10^{20}$	19.39	3.0
13	72	MRSA	SCy-Le	660	34.60	225.0	$2.07\cdot 10^{20}$	19.43	2.0
14	71	MRSA (Biofilm)	NB2	638	16.10	750.0	$7.75\cdot 10^{20}$	19.89	0.7
15	73	*S. aureus*	Curcumin	405	65.14	20.0	$4.09\cdot 10^{20}$	19.65	4.0
16	74	*S. aureus*	Curcumin	460	65.14	20.0	$4.09\cdot 10^{20}$	20.61	3.0
17	75	*S. aureus*	ICG	808	40.73	411.0	$2.19\cdot 10^{21}$	20.49	5.6
18	75	*S. aureus*	ICG	808	40.73	82.0	$4.38\cdot 10^{20}$	19.79	2.5
19	75	*S. aureus*	ICG	808	40.73	247.0	$1.32\cdot 10^{21}$	20.27	3.4
20	75	*S. aureus*	ICG	808	40.73	411.0	$2.19\cdot 10^{21}$	20.49	5.2
21	76	*S. aureus*	ICG	809	40.73	84.0	$7.19\cdot 10^{19}$	19.00	1.3
22	76	*S. aureus*	ICG	809	40.73	84.0	$1.08\cdot 10^{20}$	19.18	1.3
23	77	*S. aureus*	ICG	810	37.82	31.2	$1.94\cdot 10^{20}$	19.43	3.0
24	78	*S. aureus*	IC-H-Me2+	652	10.00	5.0	$3.28\cdot 10^{16}$	16.35	3.0
25	78	*S. aureus*	IC-H-Me2+	652	10.00	5.0	$8.20\cdot 10^{16}$	16.75	7.0
26	78	*S. aureus*	IC-H-Me2+	652	10.00	5.0	$1.64\cdot 10^{17}$	17.05	7.0
27	78	*S. aureus*	IC-H-Me2+ + KI	652	10.00	5.0	$4.10\cdot 10^{15}$	15.45	3.0
28	69	*S. aureus*	MB	660	71.55	0.1	$1.66\cdot 10^{18}$	17.91	1.0
29	69	*S. aureus*	MB	660	71.55	1.5	$3.56\cdot 10^{19}$	19.24	3.0
30	69	*S. aureus*	MB	660	71.55	5.0	$1.19\cdot 10^{20}$	19.76	5.0
31	71	*S. aureus*	NB2	638	16.10	480.0	$2.48\cdot 10^{20}$	19.39	3.0
32	71	*S. aureus*	NB1	638	25.90	480.0	$3.99\cdot 10^{20}$	19.60	2.6
33	79	*S. aureus*	Photofrin	630	1.17	225.0	$8.84\cdot 10^{19}$	19.21	5.0
34	80	*S. aureus*	TLD1411	525	0.83	100.0	$5.49\cdot 10^{18}$	18.73	7.0
35	78	*S. aureus* (Biofilm)	IC-H-Me2+	652	10.00	5.0	$8.20\cdot 10^{18}$	18.75	4.0
36	81	*Streptococcus canis*	ALA	635	3.95	13.5	$2.13\cdot 10^{18}$	18.21	0.4
37	81	*Staphylococcus intermedius*	ALA	635	3.95	13.5	$1.06\cdot 10^{19}$	18.91	0.6
38	75	*Streptococcus pyogenes*	ICG	808	40.73	411.0	$2.19\cdot 10^{21}$	20.49	4.7
39	75	*S. pyogenes*	ICG	808	40.73	82.0	$4.38\cdot 10^{20}$	19.79	3.9
30	75	*S. pyogenes*	ICG	808	40.73	247.0	$1.32\cdot 10^{21}$	20.27	6.8
41	75	*S. pyogenes*	ICG	808	40.73	411.0	$2.19\cdot 10^{21}$	20.49	6.1

**Table 2:** Summary of aPDI data of gram-negative pathogens for studies in planktonic suspension and on biofilm.

S.No.	Ref	Pathogen	Photosensitizer	λ (nm)	Ext. coeff. (${\mathrm{cm}}^{-1}\;{\mathrm{mM}}^{-1}$)	Rad. exp. ($J\;{\mathrm{cm}}^{-2}$)	Photon abs. ($\mathrm{hv}\;{\mathrm{cm}}^{-3}$)	Log (photons abs. × QY)	Log red.
Gram-negative pathogens									
1	79	*Acinetobacter baumannii*	Photofrin	630	1.17	225.0	$8.84\cdot 10^{19}$	19.21	5.0
2	77	*A. baumannii*	ICG	810	37.82	31.2	$1.94\cdot 10^{20}$	19.43	3.0
3	69	*A. baumannii*	MB	660	71.55	0.4	$9.50\cdot 10^{18}$	18.67	1.0
4	69	*A. baumannii*	MB	660	71.55	2.2	$5.23\cdot 10^{19}$	19.41	3.0
5	69	*A. baumannii*	MB	660	71.55	6.0	$1.43\cdot 10^{20}$	19.84	5.0
6	82	*A. baumannii*	Toluidine blue O	460	0.04	19.2	$6.66\cdot 10^{17}$	17.76	0.7
7	69	*Cryptococcus neoformans*	MB	660	71.55	7.0	$1.66\cdot 10^{20}$	19.91	1.0
8	69	*C. neoformans*	MB	660	71.55	16.0	$3.80\cdot 10^{20}$	20.27	3.0
9	69	*C. neoformans*	MB	660	71.55	25.0	$5.94\cdot 10^{20}$	20.46	5.0
10	69	*E. coli*	MB	660	71.55	0.3	$7.13\cdot 10^{18}$	18.54	1.0
11	69	*E. coli*	MB	660	71.55	2.0	$4.75\cdot 10^{19}$	19.37	3.0
12	69	*E. coli*	MB	660	71.55	5.0	$1.19\cdot 10^{20}$	19.76	5.0
13	81	*E. coli*	ALA	635	3.95	13.5	$2.13\cdot 10^{18}$	18.21	0.1
14	83	*E. coli*	IP-H-CF32+	415	52.40	1.4	$1.49\cdot 10^{16}$	15.84	1.0
15	83	*E. coli*	IP-H-CF32+	415	52.40	1.4	$1.49\cdot 10^{17}$	16.84	7.0
16	83	*E. coli*	IP-H-Me2+	415	53.70	1.4	$1.52\cdot 10^{16}$	15.98	2.0
17	83	*E. coli*	IP-H-Me2+	415	53.70	1.4	$1.52\cdot 10^{17}$	16.98	7.0
18	83	*E. coli*	IP-H-OH2+	415	30.20	1.4	$8.57\cdot 10^{15}$	15.36	3.0
19	83	*E. coli*	IP-H-OH2+	415	30.20	1.4	$8.57\cdot 10^{16}$	16.36	7.0
20	71	*E. coli*	NB1	638	25.90	480.0	$3.99\cdot 10^{20}$	19.60	1.8
21	71	*E. coli*	NB2	638	16.10	480.0	$2.48\cdot 10^{20}$	19.39	3.0
22	78	*E. coli*	IC-H-Me2+	652	10.00	5.0	$4.92\cdot 10^{16}$	16.53	3.0
23	78	*E. coli*	IC-H-Me2+	652	10.00	5.0	$1.64\cdot 10^{17}$	17.05	7.0
24	78	*E. coli* (243)	IC-H-Me2+ + KI	652	10.00	5.0	$8.20\cdot 10^{15}$	15.75	3.0
25	78	*E. coli* (243)	IC-H-Me2+ + KI	652	10.00	5.0	$1.64\cdot 10^{16}$	16.05	7.0
26	78	*E. coli* (ATCC 2592)	IC-H-Me2+ + KI	652	10.00	5.0	$4.10\cdot 10^{15}$	15.45	3.0
27	78	*E. coli* (ATCC 2592)	IC-H-Me2+ + KI	652	10.00	5.0	$8.20\cdot 10^{15}$	15.75	7.0
28	78	*E. coli* (Biofilm)	IC-H-Me2+	652	10.00	5.0	$8.20\cdot 10^{18}$	18.75	3.5
29	69	*Klebsiella pneumoniae*	MB	660	71.55	9.0	$2.14\cdot 10^{20}$	20.02	1.0
30	69	*K. pneumoniae*	MB	660	71.55	20.0	$4.75\cdot 10^{20}$	20.37	3.0
31	69	*K. pneumoniae*	MB	660	71.55	28.0	$6.65\cdot 10^{20}$	20.51	5.0
32	81	*P. aeruginosa*	ALA	635	3.95	13.5	$1.06\cdot 10^{19}$	18.91	0.2
33	74	*P. aeruginosa*	Curcumin	460	65.14	20.0	$4.09\cdot 10^{20}$	20.61	5.0
34	75	*P. aeruginosa*	ICG	808	40.73	411.0	$1.76\cdot 10^{22}$	21.39	2.0
35	75	*P. aeruginosa*	ICG	808	40.73	82.0	$3.50\cdot 10^{21}$	20.69	1.4
36	75	*P. aeruginosa*	ICG	808	40.73	247.0	$1.06\cdot 10^{22}$	21.17	1.8
37	75	*P. aeruginosa*	ICG	808	40.73	411.0	$1.76\cdot 10^{22}$	21.39	4.7
38	76	*P. aeruginosa*	ICG	809	40.73	252.0	$5.39\cdot 10^{21}$	20.88	2.0
39	76	*P. aeruginosa*	ICG	809	40.73	252.0	$6.74\cdot 10^{21}$	20.97	2.0
40	77	*P. aeruginosa*	ICG	810	16.65	31.2	$8.52\cdot 10^{19}$	19.08	3.0
41	69	*P. aeruginosa*	MB	660	71.55	3.8	$9.03\cdot 10^{19}$	19.65	1.0
42	69	*P. aeruginosa*	MB	660	71.55	10.0	$2.38\cdot 10^{20}$	20.07	3.0
43	69	*P. aeruginosa*	MB	660	71.55	18.0	$4.28\cdot 10^{20}$	20.32	5.0
44	80	*P. aeruginosa*	NMB	525	0.47	50.0	$9.33\cdot 10^{18}$	18.79	1.0
45	80	*P. aeruginosa*	NMB	525	0.47	100.0	$1.87\cdot 10^{19}$	19.09	2.0
46	80	*P. aeruginosa*	NMB	525	0.47	150.0	$2.80\cdot 10^{19}$	19.27	6.0
47	80	*P. aeruginosa*	Porphyrin TMPyP	525	1.31	50.0	$2.59\cdot 10^{19}$	19.28	1.0
48	80	*P. aeruginosa*	Porphyrin TMPyP	525	1.31	100.0	$5.17\cdot 10^{19}$	19.58	4.0
49	80	*P. aeruginosa*	Porphyrin TMPyP	525	1.31	150.0	$7.76\cdot 10^{19}$	19.76	7.0
50	80	*P. aeruginosa*	RB	525	3.65	50.0	$7.24\cdot 10^{19}$	19.74	3.0
51	80	*P. aeruginosa*	RB	525	3.65	100.0	$1.45\cdot 10^{20}$	20.04	7.0
52	80	*P. aeruginosa*	RB	525	3.65	150.0	$2.17\cdot 10^{20}$	20.22	7.0
53	80	*P. aeruginosa*	TLD1411	525	0.83	100.0	$5.49\cdot 10^{18}$	18.73	7.0

For aPDI preclinical and clinical studies, manuscripts that identified the PS dose, wavelength, ϕ and exposure time, or *H*, and frequency of aPDI treatment session to maximize positive biological effects toward wound healing such as accelerated healing, decreased wound size, CFU log reduction, or complete healing were selected. The analysis of the effect of bacterial species types on wound healing was not assessed, as, generally, multiple bacterial species were present in preclinical and clinical wounds. A total of 35 datasets from 26 studies were included in the data analysis. The number of absorbed photons, according to Eq. (3), was determined for a single aPDI session and the entire treatment duration to calculate the cumulative value of photons absorbed over multiple exposures. As PpIX is the active PS when ALA is used, for the studies that used ALA, we used the concentration of PpIX for the calculation of photons absorbed per unit volume, which was estimated by dividing the ALA concentration by eight equal the number of ALA required to synthesis one PpIX. Table 3 shows the preclinical and clinical studies evaluating aPDI for improved wound healing. We evaluated the most frequently used dose range and radiant exposure from the data for a positive aPDI outcome and how that dose distribution changes in the presence of hard-to-treat MRSA infections or if the infection site consists of multiple pathogens. We also evaluated the number of treatments and the interval between the treatments during the entire study for the datasets in the table.

**Table 3:** Summary of aPDI data for pre-clinical and clinical studies.

S. No.	Ref	Photosensitizer	λ (nm)	Rad. exp. ($J\;{\mathrm{cm}}^{-2}$)	Ext. coeff. (${\mathrm{cm}}^{-1}\;{\mathrm{mM}}^{-1}$)	Photon abs. ($\mathrm{hv}\;{\mathrm{cm}}^{-3}$)	Frequency (days)	Cumulative photons abs. × QY ($\mathrm{hv}\;{\mathrm{cm}}^{-3}$)	Outcome
1	84	ALA	410	10	169.61	$1.67\cdot 10^{22}$	Consecutive days	$3.59\cdot 10^{23}$	^e^
2	85	ALA	618	100	1.48	$7.01\cdot 10^{22}$	Every other week for up to 10 times	$5.40\cdot 10^{23}$	^f^
3	86	ALA	630	75	5.12	$1.16\cdot 10^{23}$	Every 2 weeks, 6 treatments	$5.36\cdot 10^{23}$	^d^ ^,^ ^f^
4	87	ALA	630	75	5.12	$1.16\cdot 10^{23}$	Weekly for 3 months	$1.07\cdot 10^{24}$	^f^
5	88	ALA	630	80	5.12	$2.48\cdot 10^{23}$	Once a week for 2 weeks	$3.81\cdot 10^{23}$	^a^ ^,^ ^b^
6	89	ALA	630	80	5.12	$2.48\cdot 10^{23}$	10 times in 14 days	$9.53\cdot 10^{24}$	^d^ ^,^ ^f^
7	85	ALA	630	20	5.12	$6.19\cdot 10^{22}$	One to three sessions	$1.43\cdot 10^{23}$	^f^
8	90	ALA	630	80	5.12	$2.48\cdot 10^{23}$	Once a week up to 3 sessions	$5.72\cdot 10^{23}$	^e^
9	91	ALA	630	80	5.12	$2.48\cdot 10^{23}$	Single	$1.91\cdot 10^{23}$	
10	92	ALA	630	20	5.12	$6.19\cdot 10^{21}$	up to 3 times 1/month	$1.43\cdot 10^{22}$	^b^
11	93	ALA	630	40	5.12	$9.90\cdot 10^{22}$	Single	$7.62\cdot 10^{22}$	^a^
12	94	ALA	630	60	5.12	$9.28\cdot 10^{22}$	Single	$7.15\cdot 10^{22}$	^a^ ^,^ ^e^
13	95	ALA	635	100	3.95	$2.40\cdot 10^{23}$	1/week until the wound is healed	$7.40\cdot 10^{23}$	^f^
14	96	ALA	635	25	3.95	$3.00\cdot 10^{22}$	Single	$2.31\cdot 10^{22}$	^a^ ^,^ ^b^
15	28	ALA	650	6	5.05	$3.96\cdot 10^{18}$	Single	$2.73\cdot 10^{18}$	^b^
16	28	ALA	650	6	5.05	$2.32\cdot 10^{21}$	Single	$1.60\cdot 10^{21}$	^b^
17	28	ALA	650	6	5.05	$1.98\cdot 10^{18}$	Single	$1.37\cdot 10^{18}$	^b^
18	70	ALA	650	60	5.05	$4.73\cdot 10^{22}$	Single	$3.26\cdot 10^{22}$	^a^ ^,^ ^e^
19	97	Chlorin e6	660	160	60.39	$3.21\cdot 10^{21}$	Single	$2.05\cdot 10^{21}$	^a^ ^,^ ^e^
20	97	Chlorin e6	660	240	60.39	$9.62\cdot 10^{21}$	Single	$6.16\cdot 10^{21}$	^a^ ^,^ ^e^
21	98	Chlorin p6	660	60	60.39	$2.41\cdot 10^{21}$	Repeat daily	$4.62\cdot 10^{21}$	^g^
22	99	Dicationic Boron Dipyrromethene	610	76	52.48	$1.53\cdot 10^{22}$	2,3,5,9	$3.18\cdot 10^{22}$	^a^
23	100	MB	665	480	74.01	$5.94\cdot 10^{22}$	Single	$2.91\cdot 10^{22}$	^a^ ^,^ ^e^
24	101	Me-ALA	600	20	8.02	$1.89\cdot 10^{18}$	Single	$1.45\cdot 10^{18}$	^a^ ^,^ ^b^
25	102	Me-ALA	630	37	5.12	$1.14\cdot 10^{23}$	Single	$8.82\cdot 10^{22}$	^a^
26	103	Me-ALA	630	18	5.12	$4.46\cdot 10^{22}$	Twice a day (10 days). Treatments every 3-week	$2.74\cdot 10^{24}$	^f^
27	104	MB	660	70	71.55	$5.20\cdot 10^{21}$	0, 3, 5, 10	$1.28\cdot 10^{22}$	
28	105	MB	660	150	71.55	$1.78\cdot 10^{21}$	Single	$8.73\cdot 10^{20}$	^a^
29	80	NMB	525	100	0.47	$6.22\cdot 10^{19}$	Single	$4.11\cdot 10^{19}$	
30	106	Phenothiazin derivative	660	24	34.00	$1.35\cdot 10^{19}$	Single	$1.35\cdot 10^{18}$	^a^ ^,^ ^e^
31	80	porphyrin TMPyP	525	100	1.31	$1.72\cdot 10^{20}$	Single	$1.28\cdot 10^{20}$	
32	107	RLP068/Cl	630	60	0.04	$2.17\cdot 10^{19}$	Twice or thrice a week	$5.35\cdot 10^{19}$	^a^
33	80	RB	525	100	3.65	$4.82\cdot 10^{20}$	Single	$3.67\cdot 10^{20}$	^a^
34	80	TLD1411	525	100	0.83	$1.10\cdot 10^{20}$	Single	$1.08\cdot 10^{20}$	
35	108	toluidine-O blue	685	4.5	7.5	$3.80\cdot 10^{19}$	Single	$3.27\cdot 10^{19}$	d,^e^^,^^g^

a CFU reduction.

b Improved wound closure rate.

c Improved epithelialization and keratinization of skin layers.

d Reduced inflammation, erythema.

e Reduced wound/ulcer size.

f Complete wound/ulcer healing/closure.

g Pro-angiogenic, neo-angiogenic effect.

For evaluating PBM, the search included studies that reported wavelength, irradiance ϕ, and exposure time, or radiant exposure H, with the frequency and duration of therapy and the clinical endpoints. A total of 14 studies with 28 datasets were included for PBM analysis in preclinical and clinical cases. To compare the number of photons absorbed by the PS to achieve a beneficial aPDI effect with PBM tissue response, it is assumed that CCO is the primary chromophore for the latter following the works of Karu^57^ and Hamblin^109^ and their teams. As for Table 3, the number of absorbed photons, according to Eq. (3), was determined for a single aPDI session, and the entire treatment duration was calculated to get the cumulative value of photons absorbed in the treatment. The CCO’s extinction coefficients, ϵ(λ),^110^ and concentrations^111^ were also obtained. Table 4 summarizes the publications investigating PBM parameters for wound closure in various preclinical and clinical studies. Clinical studies were reviewed by Zein et al.,^126^ with therapeutic doses listed for the irradiance, radiant exposure, and wavelength, as well as repeat exposure frequency if applicable; however, there was no absolute comparison between the different aPDI protocols. Similar to Table 3 for aPDI studies, we evaluated the most frequently used dose range and radiant exposure for a positive PBM outcome. We also evaluated the number of treatments and the interval between the treatments during the entire study for the datasets in the table. The dose distribution range was evaluated separately for positive, no effect, and negative effects of PBM.

**Table 4:** Summary of pre-clinical PBM in wounds.

S.No.	Ref	Wound type	λ (nm)	Rad. exp. ($J\;{\mathrm{cm}}^{-2}$)	Photon absorbed ($\mathrm{hv}\;{\mathrm{cm}}^{-3}$)	Frequency (days)	Cumulative photons ab. ($\mathrm{hv}\;{\mathrm{cm}}^{-3}$)	Outcome
1	112	Full-thickness wounds in rats	514	10.00	$7.50\cdot 10^{13}$	3 times per week	$2.25\cdot 10^{14}$	^b^
2	112	Full-thickness wounds in rats	514	20.00	$1.50\cdot 10^{14}$	3 times per week	$4.50\cdot 10^{14}$	^b^
3	112	Full-thickness wounds in rats	514	30.00	$2.25\cdot 10^{14}$	3 times per week	$6.75\cdot 10^{14}$	^b^
4	112	Full-thickness wounds in rats	514	40.00	$3.00\cdot 10^{14}$	3 times per week	$9.00\cdot 10^{14}$	^b^
5	112	Full-thickness wounds in rats	514	60.00	$4.50\cdot 10^{14}$	3 times per week	$1.35\cdot 10^{15}$	^b^
6	112	Full-thickness wounds in rats	514	80.00	$6.00\cdot 10^{14}$	3 times per week	$1.80\cdot 10^{15}$	^h^
7	112	Full-thickness wounds in rats	514	100.00	$7.50\cdot 10^{14}$	3 times per week	$2.25\cdot 10^{15}$	^i^
8	112	Full-thickness wounds in rats	514	120.00	$9.00\cdot 10^{14}$	3 times per week	$2.70\cdot 10^{15}$	^i^
9	112	Full-thickness wounds in rats	514	140.00	$1.05\cdot 10^{15}$	3 times per week	$3.15\cdot 10^{15}$	^i^
10	113	Incision on buccal mucosa in rats	632	1.00	$3.69\cdot 10^{12}$	1st day, 1st and 2nd day, 1st and 3rd day, and continuous 3 days (40 s each)	$1.11\cdot 10^{13}$	^h^
11	114	Incision in rabbits	633	2.20	$8.12\cdot 10^{12}$	14 days (twice each day for 3 min)	$2.27\cdot 10^{14}$	^h^
12	114	Incision in rats	633	2.20	$8.12\cdot 10^{12}$	14 days (twice each day for 3 min)	$2.27\cdot 10^{14}$	^h^
13	114	Incision in rats	633	4.50	$1.66\cdot 10^{13}$	14 days (twice each day for 3 min)	$4.65\cdot 10^{14}$	^h^
14	115	Rabbit surgical incision	635	2.20	$8.15\cdot 10^{12}$	7 daily	$5.71\cdot 10^{13}$	^c^
15	116	Full-thickness wounds in dogs	635	1.13	$4.17\cdot 10^{12}$	3 times a week for 32 days	$5.00\cdot 10^{13}$	^h^
16	117	Full-thickness wounds in mice	635	1.00	$3.71\cdot 10^{12}$	Single	$3.71\cdot 10^{12}$	^e^
17	117	Full-thickness wounds in mice	635	2.00	$7.41\cdot 10^{12}$	Single	$7.41\cdot 10^{12}$	^e^
18	117	Full-thickness wounds in mice	635	10.00	$3.71\cdot 10^{13}$	Single	$3.71\cdot 10^{13}$	^e^
19	117	Full-thickness wounds in mice	635	50.00	$1.85\cdot 10^{14}$	Single	$1.85\cdot 10^{14}$	^i^
20	118	Rabbit ulcer	650	1.00	$3.79\cdot 10^{12}$	30 times every 48hrs	$1.14\cdot 10^{14}$	^e^
21	119	Chronic venous ulcers	660	3.00	$1.16\cdot 10^{13}$	30, 60, and 90 days (30 s on each $5\;{\mathrm{cm}}^{2}$ area)	$3.47\cdot 10^{13}$	^b^
22	120	Rat skin	670	4.00	$1.56\cdot 10^{13}$	10 daily	$1.56\cdot 10^{14}$	^c^
23	121	Mice surgical incision	670	3.60	$1.41\cdot 10^{13}$	24 and 48 hrs after injury	$2.82\cdot 10^{13}$	^c^,e
24	121	Mice burn wounds	670	3.60	$1.41\cdot 10^{13}$	5 daily	$7.04\cdot 10^{13}$	^b^
25	122	Mice incision	670	30.00	$1.17\cdot 10^{14}$	24, 48, 96, 120, 144, 168 hrs after injury	$5.86\cdot 10^{14}$	^c^ ^,^ ^d^
26	123	3-cm sutured abdominal incision	808	0.90	$1.59\cdot 10^{12}$	5 days, twice daily, 6 min each	$1.59\cdot 10^{13}$	^h^
27	124	Mouse skin	830	40.00	$7.27\cdot 10^{13}$	5 daily	$3.63\cdot 10^{14}$	^e^
28	125	Bilateral flank ovariectomy in dogs	980	5.00	$9.65\cdot 10^{12}$	5 days daily	$4.83\cdot 10^{13}$	^h^

a CFU reduction.

b Improved wound closure rate.

c Improved epithelialization and keratinization of skin layers.

d Reduced inflammation, erythema.

e Reduced wound/ulcer size.

f Complete wound/ulcer healing/closure.

g Pro-angiogenic, neo-angiogenic effect.

h No/statistically insignificant effect.

i Reduced healing rate.

We also looked at studies evaluating the efficacy of PBM in infected wounds; however, the scope of our analysis is limited by the lack of studies reporting both the biological effects of PBM and log reduction after light exposure in infected wounds.

We evaluated the dose differences among *in vitro* aPDI, *in vivo* aPDI, and *in vivo* PBM to evaluate the dose gap between aPDI and PBM.

## 3 Results

**Fig. 2:**
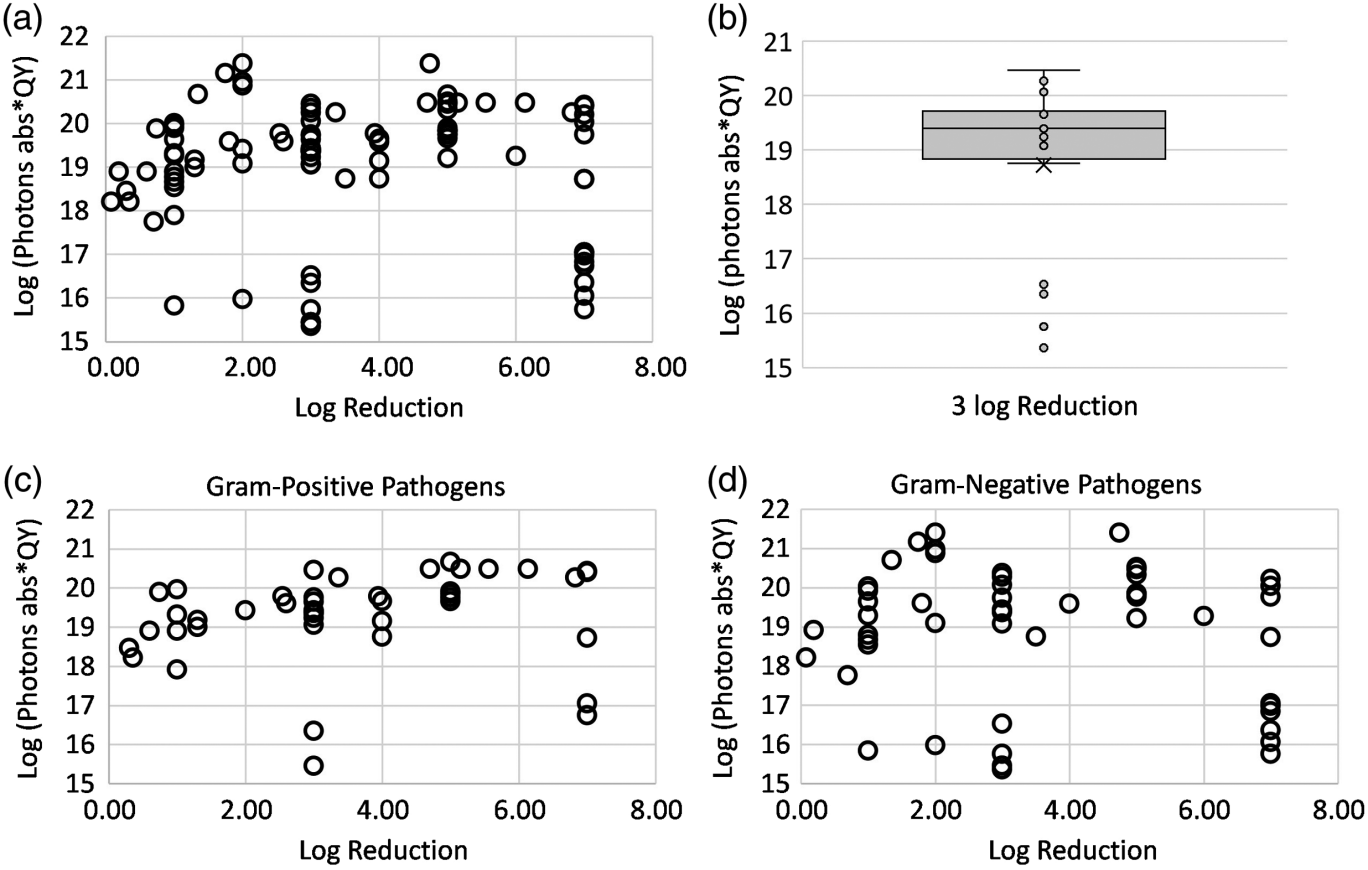
(a) Log reduction versus log of the product of photons absorbed and quantum yield or log (photons abs × QY) for all bacterial species *in vitro*. (b) Spread of the log (photons abs × QY) for all bacterial species for the inactivation threshold value of 3 log reduction. Log reduction versus log (photons abs × QY) for (c) gram-positive bacterial species *in vitro* and (d) gram-negative bacterial species *in vitro*.

Tables 1 and 2 compile the literature for various PS and microbial targets for gram-positive and gram-negative pathogens in planktonic solutions. In 94 datasets of aPDI efficacy in planktonic solution, photons absorbed per unit volume to cause disinfection or $\geq 3\;\log_{10}$ reduction ranged from $4.10\cdot 10^{15}\;\mathrm{hv}\;{\mathrm{cm}}^{-3}$ to $1.76\cdot 10^{22}\;\mathrm{hv}\;{\mathrm{cm}}^{-3}$. Figure 2(a) shows the plot of log reduction in bacteria as a function of $\log_{10}$ transformed total cytotoxic dose considering the quantum yield of the PS, and Fig. 2(b) shows the distribution of the same for only 3 log reduction in the pathogen population. The mean number of photons absorbed per unit volume considering the quantum yield of the PS required to cause 3 log reduction *in vitro* was $5.45\cdot 10^{19}\;\mathrm{hv}\;{\mathrm{cm}}^{-3}$. Figures 2(c) and 2(d) show the dose response for gram-positive and gram-negative bacteria, respectively.

**Fig. 3:**
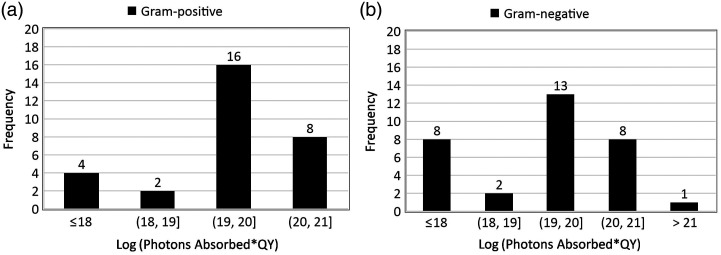
Frequency histograms showing the frequency of ≥3 log reduction as a function of log (photons absorbed × singlet oxygen quantum yield) for (a) gram-positive and (b) gram-negative bacterial species.

Figure 3 shows the frequency histograms plotted to assess the photons absorbed range distribution across the datasets for ≥3 log reduction in the pathogen population as reported in *in vitro* studies with gram-positive and gram-negative species. As indicated by the histograms, ≥3 log reduction or disinfection was achieved for a dose considering the quantum yield of the PS was in the range of $10^{19}$ to $10^{20}\;\mathrm{hv}\;{\mathrm{cm}}^{-3}$.

**Fig. 4:**
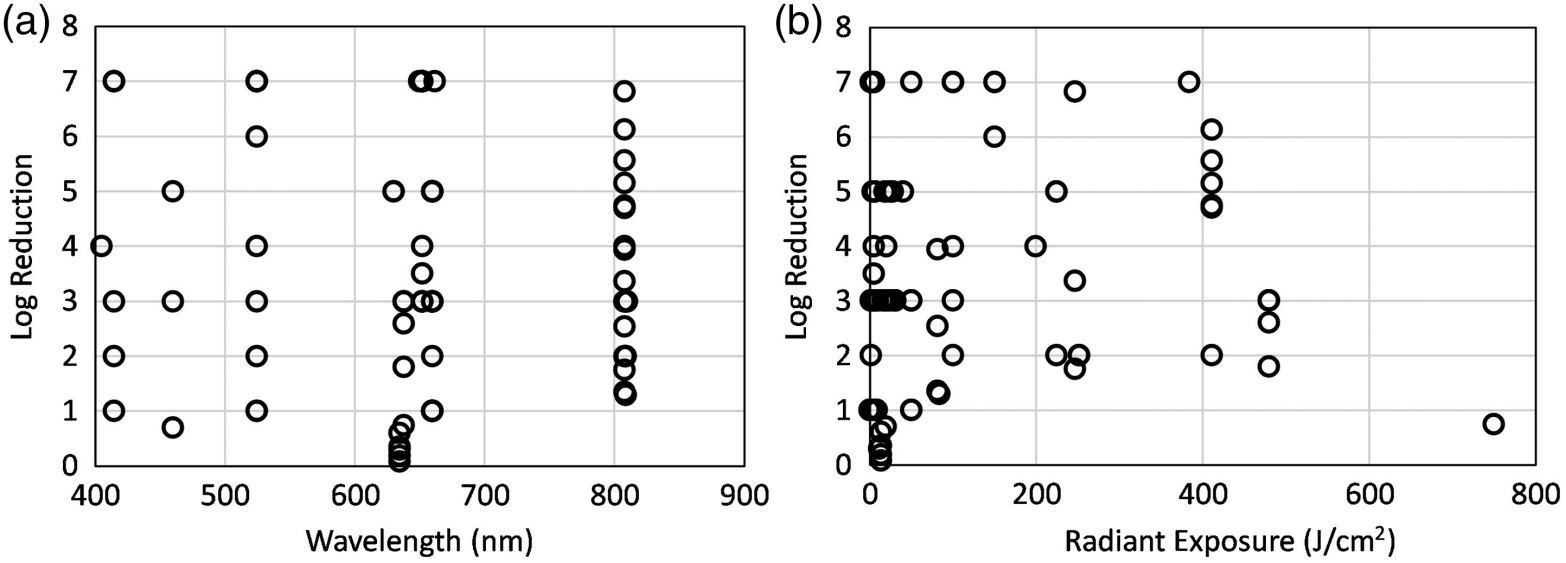
*In vitro* log reduction of all the bacterial species as a function of (a) wavelength and (b) radiant exposure.

Figure 4(a) shows the relationship between irradiation wavelength and log reduction in the pathogen population. Irradiation wavelength was not a determinant of log reduction as no correlation was seen between the two. Like wavelength, radiant exposure was also not a determinant of log reduction [Fig. 4(b)].

**Fig. 5:**
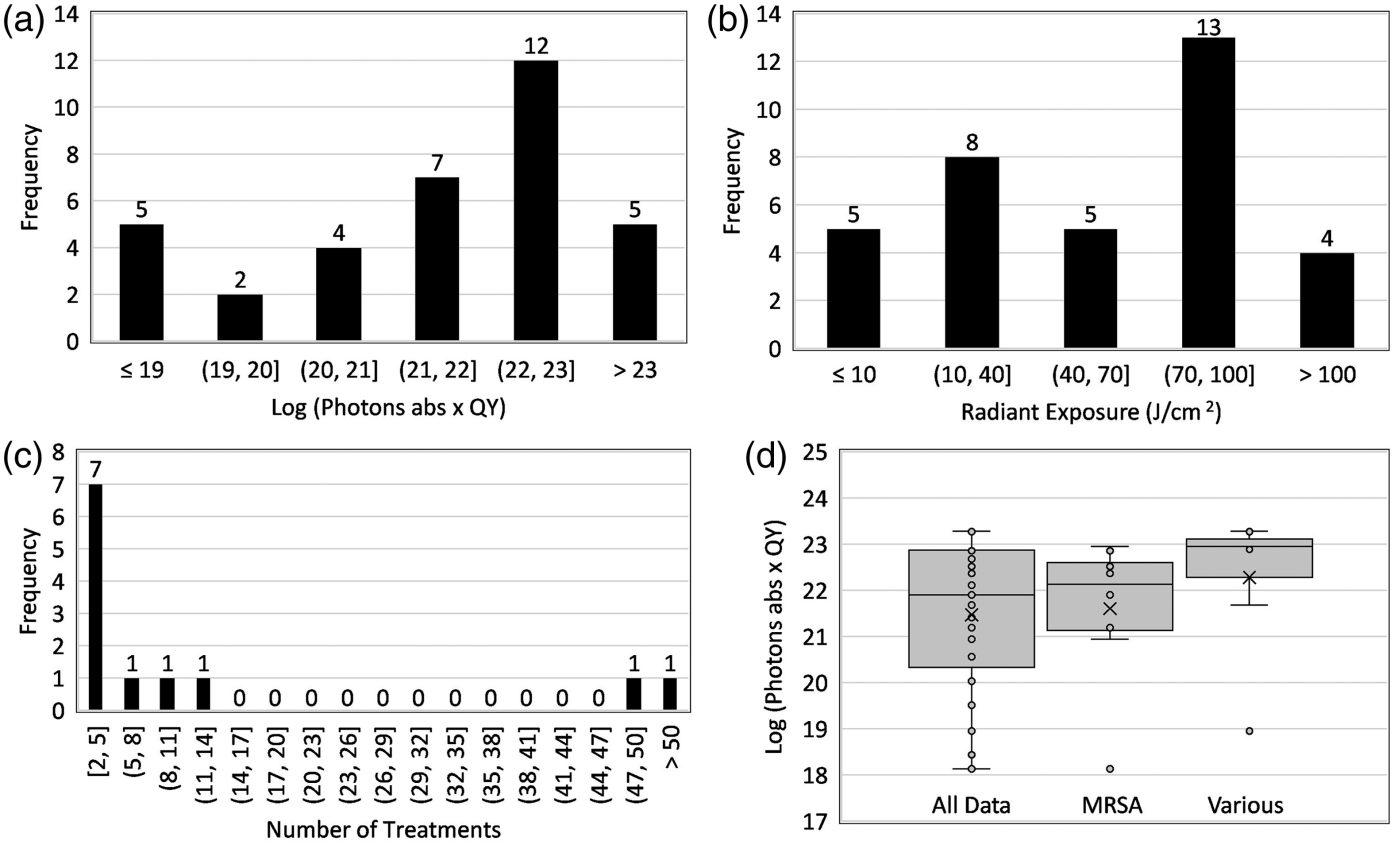
Frequency histograms showing the number of pre-clinical/clinical studies with positive outcomes from aPDI as a function of (a) log (photons absorbed × quantum oxygen yield), (b) radiant exposure ($J\;{\mathrm{cm}}^{-2}$), and (c) number of treatments (excluding single treatment). (d) The log (photons absorbed × quantum oxygen yield) for all the studies, with MRSA infection and with multiple pathogens (more than 2) on the wound site.

Table 3 shows the pre-clinical and clinical studies of aPDI-log reduction not reported in the literature for clinical cases. All the 30 datasets considered for the review reported a positive outcome of aPDI such as faster reduction in the wound area and enhanced re-epithelialization. Figure 5(a) shows the frequency histograms of the $\log_{10}$ transformed dose considering the quantum yield of the PS with the majority lying in the range of 22 to 23. Figure 5(b) shows the frequency histogram of radiant exposure reported in the studies. The majority of studies reporting a benefit from aPDI had radiant exposure in the range of 70 to $100\;J\;{\mathrm{cm}}^{-2}$. Figure 5(c) shows the number of treatments employed in the studies (excluding studies with single treatment). Of the 35 datasets, 15 performed multiple aPDI treatments during the full study period. Although the majority of the studies performed treatment in the range of two to five treatments, on the rarity, >20 treatments were also performed. Figure 5(d) shows the distribution of the $\log_{10}$ transformed dose considering the quantum yield of the PS for all datasets, just for MRSA infections and infections consisting of various pathogen species.

**Fig. 6:**
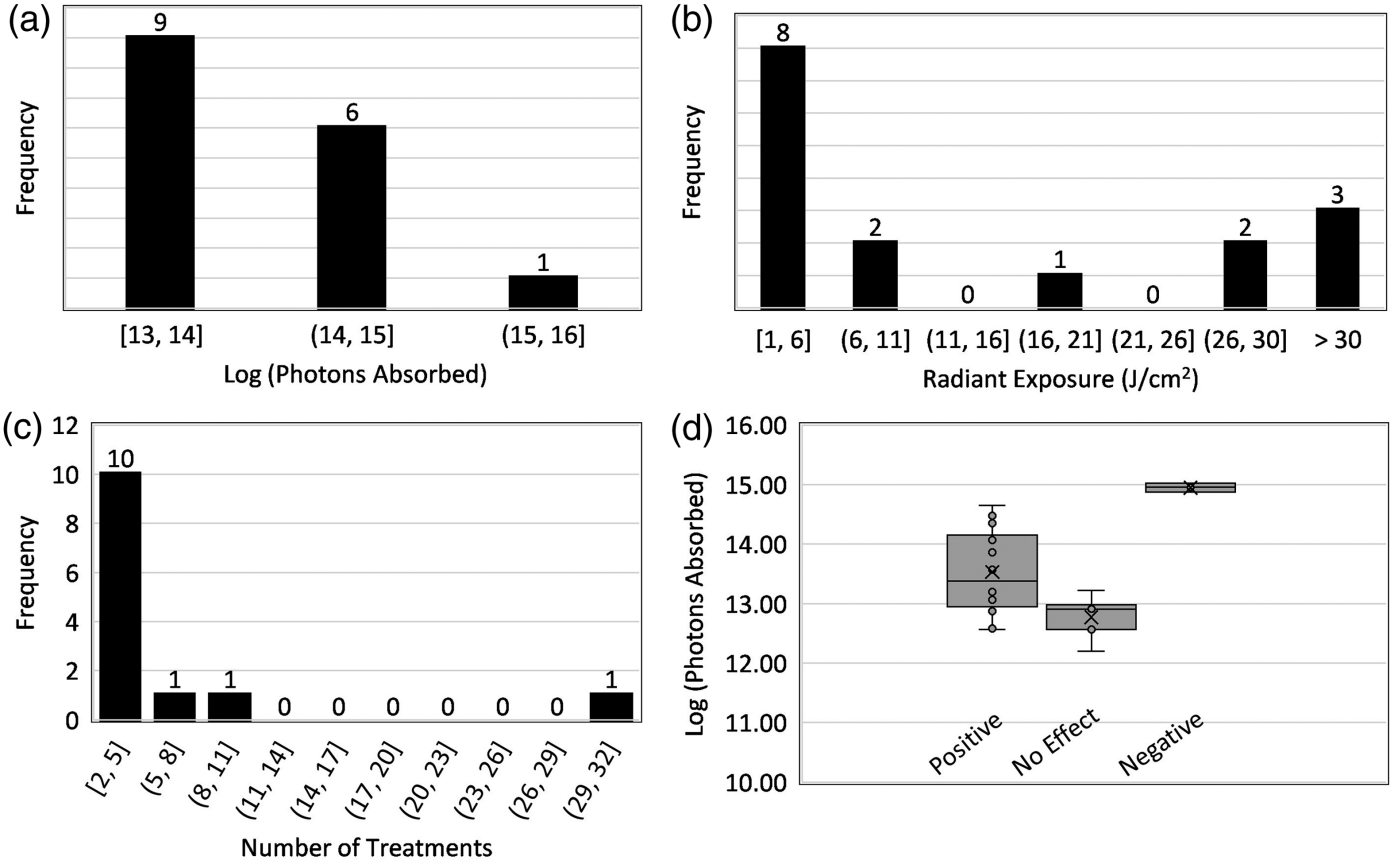
Frequency histograms showing the number of pre-clinical PBM studies with positive outcomes as a function of (a) log (photons absorbed), (b) radiant exposure [$J\;{\mathrm{cm}}^{-2}$], and (c) number of treatments (excluding single treatment). (d) The log (photons absorbed) for the studies with positive effect, no effect, and negative effect of PBM.

Table 4 shows the 28 datasets from 14 studies that evaluated the efficacy of PBM therapy *in vivo* in pre-clinical and clinical studies. From the selected datasets, 16 reported a positive biological outcome following PBM, whereas eight reported no effect on wound healing or a statistically insignificant positive effect. Four datasets showed an inhibitory response of PBM on wound healing. The most frequently reported positive biological effects were faster reduction in wound area or improved wound healing rate and enhanced re-epithelialization. The absorbed photon doses were calculated considering CCO as a dominant absorber with single exposure sessions ranging from $1.80\cdot 10^{12}$ to $9\cdot 10^{14}\;\mathrm{hv}\;{\mathrm{cm}}^{-3}$. Figure 6(a) shows a dose histogram for only the studies that reported a positive outcome post-PBM treatment. Radiant exposure, the energy delivered per unit area, ranged from 0.9 to $40\;J\;{\mathrm{cm}}^{-2}$, with the majority lying below $6\;J\;{\mathrm{cm}}^{-2}$, as shown in Fig. 6(b). The frequency histogram shows the number of treatments employed during the full study (excluding studies with single treatment) with a majority of the studies performing two to five treatments. Figure 6(d) shows the dose distribution separated for studies with positive, no effect, and negative outcomes.

A predominant wavelength range of 630 to 680 nm was employed in 16 of the 28 datasets, which may indicate the significance of this specific range in PBM applications. This range of wavelengths is within the tissue optical window, presenting penetration depth in the low mm range, depending on the skin type and within the absorption spectra of CCO, affecting PBM by stimulating biological processes due to absorption by CCO. These wavelengths also cover absorption maxima of endogenous porphyrins generated by the vast majority of bacteria, including the most abundant Propionibacterium acnes and other resident skin propionibacteria, such as *Propionibacterium granulosum*, *Propionibacterium avidum*, and *Propionibacterium humerusii*,^127^ which may help in controlling the skin bacterial population and regulating the skin’s inflammatory response to aid in wound healing. Nussbaum et al.^128^ reported that finding sterile wounds following delivery of $20\;J\;{\mathrm{cm}}^{-2}$ at 635 nm was statistically significantly higher compared with unirradiated wounds with an odd ratio of 21.5, pointing to the importance of endogenous porphyrin in the tissue cultures. This was, however, also associated with the lowest normal skin flora, largest expansion of the wound size, and slowest wound closure rate.

The effect of PBM in infected wounds was evaluated in three studies, which reported a log reduction following the PBM therapy; however, only two reported the biological impact of light on wound healing and the log reduction in bacteria. The photons absorbed during a single exposure ranged from $1.19\cdot 10^{13}$ to $2.29\cdot 10^{15}\;\mathrm{hv}\;{\mathrm{cm}}^{-3}$. The radiant exposure displayed a wide range from 3 to $288\;J\;{\mathrm{cm}}^{-2}$. Plattfaut et al.^129^ demonstrated a prolonged exposure of 2 h and utilizing light emitting diode (LED) light at 455 nm. This provides much shallower tissue penetration compared with the 630- to 680-nm range while achieving a 2.94 log reduction in human skin wounds.

**Fig. 7:**
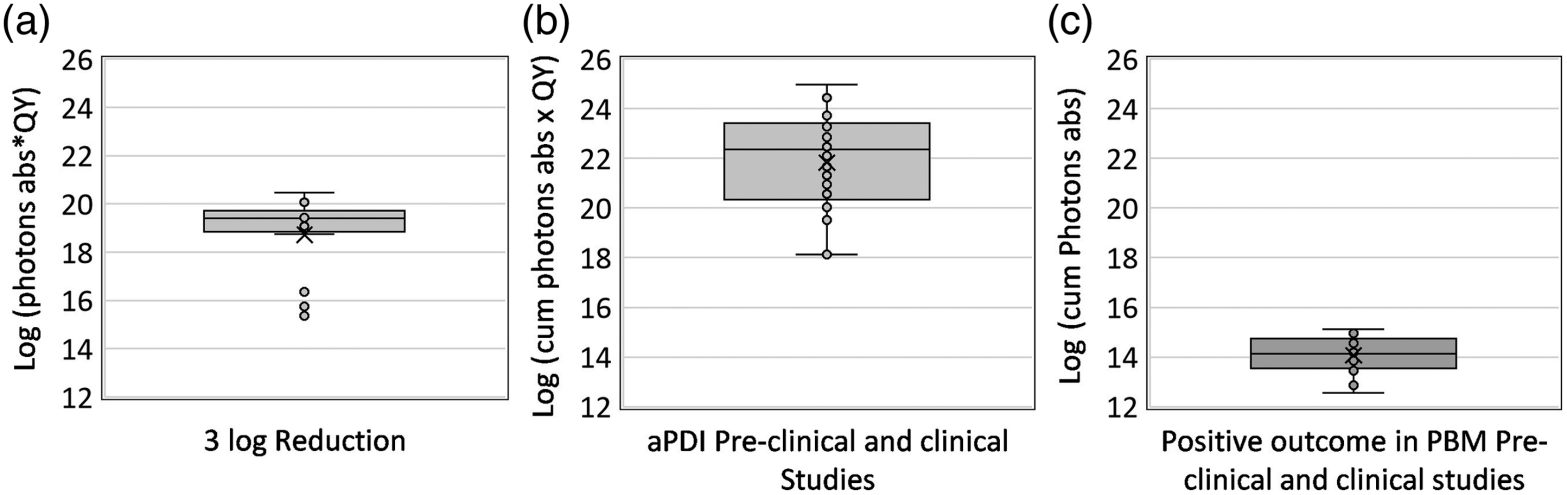
(a) The range of log (photons absorbed × QY) for the studies with 3 log reduction in pathogen population, (b) the range of log (cumulative photons absorbed × QY) for the studies that have shown positive outcome post-aPDI, and (c) the range of log (cumulative photons absorbed) for the studies that have shown positive outcome post-PBM in pre-clinical and clinical studies.

Although there was a difference of three orders of magnitude between the dose required for a 3 log reduction *in vitro* and a positive outcome of aPDI *in vivo* studies, there was a difference of eight orders of magnitude between a positive outcome *in vivo* between aPBI and PBM studies, as shown in Fig. 7.

## 4 Discussion

### 4.1 Insights and Implications

Sabino et al.^69^ (Table 1, row 30) showed a 5 log reduction in *S. aureus* population in planktonic solution when irradiated with $5\;J\;{\mathrm{cm}}^{-2}$ radiant exposure at 660 nm in the presence of 100  μM concentration of MB. The ROS dose rate, i.e., the ROS generated per second, representing the actual cytotoxic dose rate, was calculated to be $1.92\;\mu \mathrm{mol}\;s^{-1}$. Li et al.^95^ (Table 3, row 20) showed complete control and non-recurrence (for 9 months) of an ulcer in a patient with infected diabetic foot ulcer after PDT delivered by irradiating the ulcer weekly with $100\;J\;{\mathrm{cm}}^{-2}$ radiant exposure at 635 nm in the presence of 20% ALA. The ROS dose rate in this case was $244\;\mu \mathrm{mol}\;s^{-1}$. Although the bacterial load reduction was not measured in this study, a transition from infected to healed wound indicates a reduction in the infection. In both cases discussed above, the ROS dose rates were high enough to overwhelm the ROS quenching activity of the microbes,^130^ effectively reducing bacterial load or affecting a positive biological outcome. The inactivation of bacteria at such a high ROS dose rate is also an indicator of less likelihood of developing tolerance in the bacteria, which requires continuous low-dose aPDI.

As shown in the dose–response curve in Fig. 2(a), a weak correlation was found between the cytotoxic dose and log reduction. A weak positive correlation was seen for gram-positive bacteria [Fig. 2(c)], indicating that the log reduction increases as the cytotoxic dose increases. By contrast, a weak negative correlation was observed for the gram-negative species [Fig. 2(d)]. This may be due to the non-traditional PSs such as $\mathrm{IC}\text{─}H\text{─}{\mathrm{Me}}^{2+}$ (5,15-bis(1,3-dimethylimidazol-2-yl)chlorinate) that have shown high log kill for low PS concentration and low radiant exposure for both gram-positive and gram-negative pathogens (row 25, Table 1 and row 23, Table 2). In addition, the outer membrane of gram-negative bacteria acts as an additional barrier making it more challenging for PS to reach the target sites within the bacteria to disrupt the cellular processes, resulting in low log reduction even at high doses. Figure 3 shows that ≥3 log reduction, or disinfection, was achieved for a log-transformed dose, considering the quantum yield of the PS was in the range of 19 to 20, which was consistent for both gram-positive [Fig. 3(a)] and gram-negative bacterial species [Fig. 3(b)]. A log reduction of 3 is required to obtain approval for silver-containing wound covers^131^ to achieve disinfection in the wounds, thus containing the inflammatory response of the body and to get to the next stage of wound healing. The mean number of photons absorbed per unit volume, considering the quantum yield of the PS required to cause 3 log reduction *in vitro*, was $5.45\cdot 10^{19}\;\mathrm{hv}\;{\mathrm{cm}}^{-3}\;$. This could not be calculated for the pre-clinical and clinical cases as the log reduction was not reported as an outcome in most cases.

For *in vivo* aPDI studies, the log-transformed dose range was three orders of magnitude higher than the dose required for disinfection during *in vitro* studies. This is expected as factors such as distribution of PS, availability of oxygen, and presence of eschar in the wound site affect the absorption of photons and release of ROS to cause cell kill. It is also to be considered that we estimated the concentration of PpIX in the *in vivo* aPDI studies that used ALA as the PS by dividing the concentration of ALA by 8. This is an approximation and possibly an overestimation of the PpIX concentration, but we do not know the biosynthesis rate of the actual bacteria to have a more accurate calculation for this; hence, they may change the dose range for *in vivo* aPDI cases.

Passarella and Karu^132^ hypothesized that although CCO is the dominant PBM photo absorber, the roles of other factors such as the presence of ROS and local increase in temperature of the chromophores cannot be ignored. ROS, such as superoxide and singlet oxygen species, can be generated in cells due to high fluence irradiation, which can cause bioeffects such as keratinocyte proliferation *in vitro*. Local heating caused by light absorption may inhibit or activate some enzymes, resulting in biomodulation of the microbes’ and mammalian cell metabolisms. Given that the thermal relaxation time of 1  μm-sized objects is ∼1  msec, local heating of the microbe is not to be expected for continuous wave exposures commonly used even at kHz intensity modulations. Nevertheless, temporal modulation of the irradiance in the low kHz regime was shown to cause increased microbe proliferation, particularly for 810-nm NIR exposure of *P. aeruginosa*, whereas the effect was less for *S. aureus* and *E. coli*.^133^

For PBM studies, the mean number of photons absorbed per unit volume to affect positive wound healing, considering CCO as the dominant absorber *in vivo*, was $9.41\cdot 10^{13}\;\mathrm{hv}\;{\mathrm{cm}}^{-3}$ (calculated from Table 4, column 6). Considering the multiple number of treatments delivered during the entire study duration, the mean cumulative number of photons absorbed per unit volume to affect positive wound healing was slightly higher than the single exposure at $3.16\cdot 10^{14}\;\mathrm{hv}\;{\mathrm{cm}}^{-3}$(calculated from Table 3, column 8). There was an observable dose difference in the studies that showed positive, no effect or statistically insignificant effect, and negative effect, with low doses being ineffective in bringing about healing and high doses causing inhibition of the healing process [Fig. 6(d)]. The mean cumulative dose to cause an inhibitory effect was an order higher than the positive dose at $2.05\cdot 10^{15}\;\mathrm{hv}\;{\mathrm{cm}}^{-3}$ (calculated from Table 3, column 8), indicating the presence of an upper dose limit for wound healing also predicted by the biphasic tissue response. Identifying aPDI treatment conditions so that the light dose does not exceed this limit is required so as not to delay wound healing, which would become a detrimental side effect of the therapy.

### 4.2 Challenges

Clinical translation of aPDI is hindered by the wide variability in the tissue response. For the reported log reduction in pre-clinical and clinical aPDI cases (Table 2), the reduction in the viable counts ranged from 1 to 6 log reduction with a relatively lower response from resistant strains. Grinholc et al.^134^ also showed that the aPDI effect was strain-dependent and ranged from a 0 to 3 log reduction in viable counts for protoporphyrin diarginate, a PpIX derivative in 40 MRSA and 40 Meticillin-Sensitive Staphylococcus aureus (MSSR) strains. The biological cause for this variability in aPDI responsivity is unclear.

One common concern when developing novel antimicrobial strategies is the induction of resistance or tolerance to the therapy. Factors leading to resistance or susceptibility to aPDI include oxidative stress detection and neutralization, stress response regulators, DNA repair, and the membrane properties determining uptake (external, intracellular uptake, or active transport). The latter was recently reviewed.^134^ However, how these different factors render microbes sensitive to an aPDI by a particular PS is unknown in most cases.

A 2017 review^135^ suggested that given the ROS-dependent mechanisms of action of aPDI, which indiscriminately oxidizes proteins and lipids, they are unlikely to induce microbial resistance. However, the number of surviving microbes may have been too low for the statement to be conclusive. Moreover, it is well established that $H_{2}O_{2}$ has a mutagenic potential mbox,^136^ whereas the mutagenic potential for O21 appears weaker, as long as the PS is not within 10^th^ of nm from the DNA. In mammalian cells, PS localization is typically far from the nucleus, reducing mutagenic risk, but microbial DNA is within the reach of some longer-lived ROS, particularly for $H_{2}O_{2}$. Rapacka-Zdonczyk et al.^137^ showed the ability to develop tolerance in multiple clinical MRSA and MSSA strains after 15 successive aPDIs mediated by either RB, 5,10,15,20-tetrakis(1-methyl-4-pyridinio) porphyrin tetra (p-toluene sulfonate) (TMPyP) or NMB while regrowing bacteria directly from the planktonic solution. Tolerance was observed upon sub-lethal RB-mediated aPDI, which remained stable in the surviving fraction. The recombinant DNA repair protein recA appears to have a central role in developing tolerance as recA-deficient *S. aureus* mutants remained sensitive under identical aPDI protocols.

Studies completed in planktonic cultures may also not be suitable to evaluate the induction of tolerance and resistance, as pointed out by Rapacka-Zdonczyk et al..^138^ Assessing induction of tolerance or resistance should be completed for bacteria in biofilm mode as it is the standard *in vivo* growth condition that will enable horizontal gene transfer.^139^ However, compared with antibiotics, it was demonstrated that resistance required continuous low-dose exposure, whereas aPDI is designed to be delivered as a short bolus-like procedure.

### 4.3 Pathways to Optimization

As mentioned before, aPDI and PBM are photonics-based techniques that have significant interaction between them; hence, to utilize both techniques in a complimentary way for promoting wound healing, approaches for combining the two are needed. From Figs. 5 and 6, it is evident that there is a photon density difference of eight orders of magnitude between effective aPDI (*in vivo*) and PBM. This photon density gap needs to be minimized or eliminated for wound disinfection without delaying or interfering with wound healing and closure. The development of new PSs that have shown high log kill for low photon density has shown promise to achieve this.^78^^,^^83^ Reducing the photon density gap may also be possible by utilizing the endogenous porphyrins of the bacteria to generate ROS for microbial inactivation and reducing the burden on the PS to achieve disinfection. Studies have shown that shorter wavelengths between 400 and 500 nm effectively kill bacteria^140^ using the endogenous porphyrins generated by the bacteria, given the porphyrin’s up to 100 times higher molar extinction coefficient at these wavelengths, achieving the aPDI absorbed dose at lower fluences. Furthermore, the absorption coefficient of CCO is ∼10 times higher at these wavelengths,^141^ which would require lower irradiance to cause inhibitory PBM effects and thus may help reduce the adverse effects of absorption of high irradiation in the normal cells.

Another strategy would be to interleave aPDI and PBM in the time domain, alternating the two effects at their most effective activation wavelength and irradiances. However, one needs to know the effects of the washout period, particularly for PBM. Most PBM protocols associated with wound healing call for 24 to 48 h repeat cycles,^142^ which may be too long for aPDI if the CFU reduction did not achieve 6 to 7 logs. Conversely, the PBM growth benefit for bacteria does not appear to extend beyond one cell cycle.^133^

One potential solution for mitigating the photon density mismatch between preferred PBM and aPDI treatment protocols could be in low-dose aPDI combined with low-dose antimicrobial therapies, representing currently a very intense research direction, which was recently reviewed multiple times.^143^^,^^144^ Repeated observations are that porphyrins, endogenous or exogenous, and MB-based low-dose aPDI in combination with antibiotics are promising against *P. aeruginosa in vitro*, independent of the microbes’ antibiotic sensitivity to antibiotics. Combinations of different antibiotics with aPDI mediated by RB, phenothiaziniums, or porphyrins can provide a synergistic effect *in vitro*; however, at present, one cannot predict the efficacy based on a particular microbe strain. In addition, Wozniak and Grinholc^144^ pointed out that most studies claiming synergism do not follow the required methodology. Nevertheless, gentamicin showed the most consistent benefit against both gram-positive and gram-negative bacteria among the antibiotics. The reported inactivation gains compared with the mono-therapies exceeded in general by 2 logs, with some reports reaching 8 logs increased inactivation.^145^

There is also a considerable push to use nanoparticles,^146^^,^^147^ and or nanocarriers^148^ in aPDI; however, given the often ill-defined PS concentrations in these nano constructs, we could not include them in the present research. The benefit of phospholipid/ethanol-based nanocarrier-mediated PS transport was recently elegantly demonstrated by Shiryaev et al.,^149^ showing that, in a clinical study comprising patients presenting with multiple antibiotic-resistant microbes, the efficacies of MB, Photosens (AlPc), and Fotoran e6 (Ce6) for wound sterilization and wound closure were improved. Augmenting aPDI with simultaneous or sequential photothermal therapy through the use of strong organic absorbers, such as Prussian Blue^150^ or metal-organic frameworks^151^ or metal-based nanocarriers,^152^ provides other avenues to reduce the overall photon density for aPDI while employing other co-therapies simultaneously. Similar to other *in vivo* studies, the majority of the nanotechnology-based aPDI studies showed accelerated wound closure compared with infected control wounds for the first week, whereas at 3 weeks, the difference in wound closure is minimal. Interested readers should consult the review of Youf et al.^153^ Other nanotechnology-independent approaches to improve aPDI efficacy are via the use of different delivery methods, including Pluronic^106^ or functionalized polydimethylsiloxane wound dressings.^154^

## 5 Conclusion

aPDI for SSI or chronic wounds can provide an antimicrobial-free therapy option, supporting the required antimicrobial stewardship and aligning with the UN’s sustainable development goals. Delivery of aPDI can be initiated independent of the microbial strains infecting the wound. However, acceptance of this therapy is limited by the uncertainty of the required delivered PS dose, radiant exposures, and the complexities around the time required to deliver the therapy. With this study, one can derive clearer guidelines on reporting the study parameters using the metric: photons absorbed by the key chromophore, PS, and CCO, per unit volume, as this metric considers the critical variables that determine the outcome of aPDI and PBM.

Maximizing the efficacy of both aPDI and PBM in wound disinfection and healing requires balancing the photon density during therapies; the approaches to achieve that were elaborated on in Sec. 4 and are summarized as:

1. Avoid aPDI excitation wavelength where CCO has a strong absorption coefficient. Ideally, the PS should have a very strong absorption coefficient wherever there is minimal CCO absorption.
2. Increasing the molar extinction of the PS by either increasing its concentration or targeting the Soret-band rather than the q-band for excitation allows to achieve the required aPDI dose of absorbed photons given the higher absorption coefficient while reducing the photon density affecting PBM.
3. A combination approach of aPDI and low-dose antimicrobial reduces the photon density required for wound disinfection while maintaining antimicrobial efficacy.
4. An approach of time multiplexing of aPDI and PBM delivery may mitigate the differential aPDI and PBM photon density, that is, a dedicated PBM therapy preceding the aPDI therapy to first stimulate the fibroblast and granulocytes prior to inhibiting them during aPDI. For this to become most effective, further studies are required to establish the wash-out time of the PBM *in vivo*.

*In vivo* studies, taking both aPDI and PBM into consideration, are urgently required.

To enable the translation of aPDI as a means to prevent, control, and accelerate the closure of infected wounds without the use of antibiotics, the industry must be placed into the position to quantitatively evaluate the efficacy of the various PSs, delivered either as organic molecules or via nanocarriers or gels; reporting of the effect size must be accompanied the applied radiant exposure, treatment wavelength, the PS’s concentration, and molar extinction coefficient at a minimum. Successful translation and commercialization will become an integral part of supporting antimicrobial stewardship.

## Data Availability

Data and spreadsheets extracting the pertinent information from the different publications to calculate the PDI and PBM threshold values will be available upon request.
